# Suicidal ideation of people living with HIV and its relations to depression, anxiety and social support

**DOI:** 10.1186/s40359-023-01177-4

**Published:** 2023-05-16

**Authors:** Yong Yu, Bangan Luo, Lulu Qin, Hongjie Gong, Yijia Chen

**Affiliations:** 1 School of Politics and Public Administration, Guangxi Normal University, Guilin, 541006 China; 2 Department of Mental Health, Brain Hospital of Hunan Province, The Second People’s Hospital of Hunan Province, Changsha, 410007 China; 3 Department of Social Medicine and Health Management, School of Medicine, Hunan Normal University, Changsha, 410081 China; 4 Medical-Humanities Center of Hunan Normal University, Hunan Normal University, Changsha, 410081 China

**Keywords:** PLWH, Suicide ideation, Anxiety, Depression, PSSS, Intermediary effect

## Abstract

**Background:**

The HIV/AIDS (human immunodeficiency virus/acquired immune deficiency syndrome) remains a global threat to health. Suicidal ideation has been a serious public health problem among people living with HIV (PLWH). However, the suicide prevention mechanism among PLWH still unclear. This study aims to analyze the suicidal ideation and its related factors in PLWH, and further explore the relationships between suicidal ideation and depression, anxiety and perceived social support.

**Methods:**

This is a cross-sectional study. A total of 1146 PLWH were investigated by the general information questionnaire, the perceived social support scale (PSSS), the Beck scale for suicide ideation of Chinese version (BSI-CV), the generalized anxiety disorder scale-2 (GAD-2) and the patient health questionnaire-2 (PHQ-2) though the WeChat in China in 2018. By using statistical description and the binary unconditional logistic regression, we assessed the incidence of suicidal ideation and its related factors in PLWH. Besides, the intermediary effect of social support between anxiety, depression and suicidal ideation were explored by the stepwise test and Bootstrap method.

**Results:**

The incidence of suicide ideation was 54.0% (619/1146) among the PLWH in the last week or during the most serious depression. Binary logistic regression analysis results showed that the PLWH who with short time for HIV positive diagnosis (*aOR* (adjusted odd ratio) = 1.754, 95% CI (confidence interval):1.338–2.299), low monthly income (*aOR* = 1.515, 95%*CI*:1.098–2.092), other chronic diseases except HIV (*aOR* = 1.555, 95%*CI*:1.134–2.132), irregular lovers (*aOR* = 1.369, 95%*CI*:1.021–1.837), anxiety (*aOR* = 2.711, 95%*CI*:1.767–4.161), depression (*aOR* = 1.614, 95%*CI*:1.078–2.417), low PSSS (*aOR* = 2.139, 95%*CI*:1.345–3.399) had high risk of suicide ideation.The social support played a mediating role between the anxiety (the mediating effect accounted for 30.43% of the total effect), depression (the mediating effect accounted for 23.76% of the total effect) and the suicide ideation among PLWH.

**Conclusion:**

The incidence of suicide ideation of PLWH was high. Anxiety, depression, and social support are the key factors of suicide ideation of PLWH. Social support plays a partial mediating role between anxiety, depression and suicidal ideation, which provides a new approach for prevention of suicidal ideation in PLWH and should be known widely for people to prevent suicide.

## Introduction

The HIV/AIDS (Human Immunodeficiency Virus/Acquired Immune Deficiency Syndrome) remains a global threat to health. When the first PLWH (people living with HIV) reported in 1981 in USA, HIV/AIDS has spread across the world. According the report of WHO (world health organization) in 2018, there were a number of 37.9 million PLWH worldwide, and 21% of the PLWH were from the Asian-Pacific region [1]. In China, there were 1.045 million PLWH up to October 31, 2020 [2]. Consequently, the HIV/AIDS has been an important public health and social concern, and its related disease burden cannot be ignored.

Suicide ideation has been a serious public health problem among PLWH. As the ART (antiretroviral therapy) used for PLWH, the mortality of PLWH is lowed. However, the ART only plays the role of the inhibition of HIV virus replication, which is not an effective killing way of HIV virus. Besides, the ART will bring some adverse reactions to PLWH, such as mood disorder [3] and other side effects, which may lead to suicide ideation [4]. Meanwhile, though the AIDS is a controllable chronic disease by the way of ART [5], its subsequent pressure and psychological burden are quite serious without curing. Previous studies have shown that PLWH often needs to bear a lot of pressure related to their diseases, including stigma, discrimination, economic difficulties, side effects of ART treatment, unemployment and so on, leading to a negative impact on their mental health significantly (such as depression, anxiety, suicide, etc.) [6, 7].

Many studies suggest that the mediating role of social support in mental health deserves exploration. A study conducted among the elderly aged 65 and above in South Korea found that social support played a part of the intermediary role between social support and depression, which indicated that depression would indirectly affect suicide behavior by influencing the social support [8]. Some other studies have found the intermediary role of social support between depression, shame and other social psychological factors and suicide in other groups. Social support have be proved to be one of the important factors affecting PLWH's mental health from previous studies [9, 10], however, there is a lack of studies researching on the intermediary role of social support between depression, anxiety and suicide ideation in PLWH.

Suicide prevention mechanism among PLWH still unclear. In the development of suicidal behavior among PLWH, suicidal ideation is the first sign and about 70% of the PLWH will disclose their suicidal ideation in many ways before their suicide actions. According to Keiser [11] and Schlebusch [12], the rate of suicide ideation among PLWH was accounted for 39% and above, which is three times higher than that of general population. The more suicide ideation, the more suicidal behavior. Considering the bad condition of mental health among PLWH (especially the anxiety, depression, social support), it is important and feasible to prevent suicide from suicide ideation by the way of anxiety, depression, social support. This study aims to assess the incidence of suicide ideation and its influencing factors among PLWH, and further explore its relationships with depression, anxiety and perceived social support through the WeChat platform PLWH in Shizhong District, Jinan City, Shandong Province, China.

## Materials and methods

### Study population and procedures

Considering the widespread use of the WeChat platform, this study has taken the PLWH users in the WeChat platform set up by the Centers for Disease Control and Prevention (CDC) in Shizhong District, Jinan, Shandong Province, China as the research object. The WeChat platform “Lihui Space Time”, conducted in July 2014, aims to provide AIDS knowledge, psychological counseling and medication guidance for people who affected by HIV. Up to now, there are more than 140,000 users, and 90% of them are PLWH across the China. For PLWH who needs further consultation on the WeChat platform, the CDC has set up a dedicated WeChat account for further communication and exchange. As of September 1, 2018, the CDC has established three dedicated WeChat accounts, with a total of 9987 PLWH users.

Though the three WeChat accounts, we recruited respondents publicly. Inclusion criteria: 1) ≥ 18 years old; 2) HIV positive confirmed by CDC; 3) having been treated in the way of ART; 4) without cognitive dysfunction; 4) willing to accept our survey and sign the informed consent form. Exclusion criteria: 1) those who have not yet started the ART treatment after confirmed HIV positive by CDC; 2) those who are unable or unsuitable to be investigated due to serious physical or mental diseases. Finally, we successfully recruited 1166 PLWH to participate in our survey. After conducting the one-to-one network survey, a total of 1146 valid samples were obtained (1146/1166, 98.28%), which meets the sample size standard (250–400 persons) recommended by the WHO for sentinel surveillance of high-risk groups of sexually transmitted infections [13].

### Data collection and measurements

#### Socio-demographic information

Socio-demographic information collection included gender, age, nationality, education, marital status, being a student or not, living conditions, occupation, average monthly income, raising children or not, having partners or not, the way of HIV infection, being with other chronic diseases or not.

#### Social support

The perceived social support scale (PSSS) was used to investigate the social support of PLWH [14]. The PSSS contains 12 items, which are divided into three dimensions: family support, friend support and other support. The likert-7 score method was used in every item, and the sum of the scores of each item is the total score (or the score of each dimension). The total score of PSSS varies from 12 to 84, which reflects the level of social support that individuals perceive: 1) low support:12–36 points; 2) medium support:37–60 points, and 3) high support: 61–84 points. The scale has been widely used in China with good reliability and validity (Cronbach’s α = 0.840, KMO (Kaiser–Meyer–Olkin) = 0.783) [15, 16].

#### Anxiety and depression

The generalized anxiety disorder (GAD-2) and the Chinese version of the patient health questionnaire (PHQ-2) were used to screen the anxiety and depression of PLWH. The GAD-2 is often used to screen anxiety. Respondents were asked to answer the situation and frequency described in Item 1 and Item 2 in the past two weeks (Item 1: feeling nervous, anxious or impatience; Item 2: unstopped or uncontrollable worry.). There are four options in every item: not at all, for days, more than a week, almost every day. The total score of the GAD-2 ranges from 0 to 6, and the score ≥ 3 is used to be the cut-off point for clinical anxiety screening. Studies have shown that the GAD-2 has good reliability and validity in anxiety screening (Cronbach’s α = 0.87, AUC (area under the curve) = 0.91) [17].

The PHQ-2 contains two items (Item 1: lack of interest and fun in doing things; Item 2: being down in spirits, depression and hopelessness) [18]. The PHQ-2 is used to determine the change of feeling in the past two weeks. Among them, 0 score means not found, 1 score means occasional days, 2 score means more than half of the two weeks, and 3 score means almost daily. The total score of the PHQ-2 is the adding of the two items scores, which ranges from 0 to 6. According to the total score of the PHQ-2, the severity of depressive disorder can be evaluated, and score ≥ 3 is the cut-off value of depression symptom screening. The PHQ-2 has good reliability and validity (Cronbach’s α = 0.785, AUC = 0.806.)

#### Suicide ideation

The Chinese Version of Beck Scale for Suicide Ideation (BSI-CV) was used to assess the existence and severity of suicide ideation in the last week and at the time of the most severe depression [19]. BSI-CV contains 19 items, which are divided into two factors: suicidal ideation (the first 5 items) and suicidal tendency (the last 14 items) and has been proved to be with good reliability and validity of college students (Cronbach of BSI-CV α Coefficient = 0.87, Cronbach of suicide ideation sub dimension α = 0.89) [19].

According to the BSI-CV, the suicide ideation during the past week and at the time of the most severe depression is measured by the 5 items: 1) How much do you want to live? 2) How much do you want to die? 3) Are your reasons for living better than your reasons for dying? 4) How much do you want to commit suicide? 5) To what extent do you want external forces to end your life, that is, "passive suicide desire"? (For example, I hope I can sleep forever and never wake up, or die unexpectedly.). Score method is defined as follow: 1) The likert-3 score method was used in every item, and the sum of the scores of each item is the total score. The three options for the 5 items are: no (scored as 0), weak (scored as 1), moderate to strong (scored as 2). 2) If the answer to Item 4 and Item 5 is "No", it is considered that there is no suicidal ideation, others are defined to be with suicidal ideation. 3) For those who have suicidal ideation, we also continue to calculate the intensity of their suicidal ideation, that is, the total score of five items. The higher the score, the stronger the suicidal ideation. If there is no suicidal ideation, the total score of suicidal ideation is 0.

### Quality control

Firstly, all the respondents were interviewed through the WeChat one-on-one by the investigators, and each online survey was guaranteed to be conducted in a quiet and undisturbed environment. Each interview questionnaire should be verified on the spot. The items that the respondents did not answer in time or did not give a clear answer at effective time were inquired and confirmed under the basis of ensuring the compliance with ethical principles. Secondly, the investigators were trained professionally prior to administering the survey to ensure that each investigator could conduct the surveys in accordance with uniform standards and ensure standardized survey procedures. Each investigator was required to use the same instructions for each respondent.

### Statistical methods

EpiData 3.1 was used for entry into the database, and SPSS 21.0 was used for statistical analysis. The measurement data were expressed by mean ± standard deviation, the counting data were described by the number of cases and constituent ratio, and the comparison between groups was performed by Chi square (*χ*^2^) tests. Further, we take PLWH having suicidal ideation or not as the dependent variable, the HIV positive diagnosis time (1 = ≤ 35 months, 2 = > 35 months), infection way (1 = MSM (Male Have Sex With Male), 2 = Heterosexual behavior, 3 = Blood transmission/mother to child transmission), age (1 = ≤ 31 years, 2 = > 31 years), gender (1 = male, 2 = female), original residence (1 = urban, 2 = rural), nationality (1 = Han, 2 = other nationalities), marital status (1 = unmarried, 2 = married, 3 = divorced/widowed), employment (1 = unemployment, 2 = students, 3 = free employment, 4 = civil servant/private employee), single child or not (1 = yes, 2 = no), education level (1 = middle school/primary school, 2 = high school or technical secondary school, 3 = college, 4 = bachelor, 5 = master degree and above), living style (1 = Living alone, 2 = Living with others), the current average monthly income (1 = ≤ 4000 RMB, 2 = > 4000 RMB), the abuse of new drugs (1 = yes, 2 = no), other chronic diseases except HIV (1 = yes, 2 = no), the results of the last physical examination (1 = normal, 2 = abnormal), telling his/her HIV infection to family members or not (1 = yes, 2 = no), telling his/her HIV infection to friends or not (1 = yes, 2 = no), telling his/her HIV infection to others or not (1 = yes, 2 = no), having fixed lovers (1 = yes, 2 = no), anxiety (1 = yes, 2 = no), depression (1 = yes, 2 = no), social support (1 = low, 2 = medium, 3 = high) were taken as independent variable. The binary unconditional logistic regression was used to analyze the related factors of suicide ideation, and step by step test and Bootstrap method are used to test the mediation effect. In this study, *P* < 0.05 are statistically significant.

## Results

### The suicide ideation and sociodemographic characteristics of PLWH

Table 1 showed the characteristic information of the samples. As shown in Table 1, a total of 619 (54.01%) PLWH reported suicidal ideation in the past week or at the time of the most severe depression. There were statistically significance in the prevalence rate of suicide ideation among PLWH with different situation of HIV positive diagnosis time, age, registered residence, employment, living conditions, monthly income, with a fixed lover or not, with other chronic diseases except HIV or not, the result of the latest physical examination normal or not, telling their friends about HIV infection or not, depression or not, anxious or not, and different levels of social support (*P* < 0.05).

**Table 1:** Compares of suicide ideation with different sociodemographic characteristics [n (%)]

Variables	Total (n = 1146)	Suicide ideation (n = 619)	Non-suicide ideation (n = 527)	*χ*^*2*^	*P*
*Infection way*					
Male have sex with male	951 (83.0)	505 (53.1)	446 (46.9)	2.771	0.250
Heterosexual behavior	86 (7.5)	47 (54.7)	39 (45.3)		
Blood transmission/mother to child transmission	109 (9.5)	67 (61.5)	42 (38.5)		
*HIV positive diagnosis time*					
≤ 35 months	584 (51.0)	362 (62.0)	222 (38.0)	30.472	< 0.001
> 36 months	562 (49.1)	257 (45.7)	305 (54.3)		
*Age*					
≤ 31 years	634 (55.3)	366 (57.7)	268 (42.3)	7.884	0.005
> 31 years	512 (44.7)	253 (49.4)	259 (50.6)		
*Gender*					
Male	1101 (96.1)	595 (54.0)	506 (46.0)	0.009	0.926
Female	45 (3.9)	24 (53.3)	21 (45.7)		
*Original residence*					
Urban	670 (58.5)	339 (50.6)	331 (49.4)	7.582	0.006
Rural	476 (41.5)	280 (58.8)	196 (41.2)		
*Nationality*					
Han	1073 (93.6)	573 (53.4)	500 (46.6)	2.542	0.111
National minority	73 (6.4)	46 (63.0)	27 (37.0)		
*Marital status*					
Unmarried	826 (72.1)	446 (54.0)	380 (46.0)	1.450	0.484
Married	245 (21.4)	137 (55.9)	108 (44.1)		
Divorced/widowed	75 (6.5)	36 (48.0)	39 (52.0)		
*Single child or not*					
Yes	498 (43.5)	259 (52.0)	239 (48.0)	1.427	0.232
No	648 (56.5)	360 (55.6)	288 (44.4)		
*Employment*					
Unemployment	73 (6.4)	57 (78.1)	16 (21.9)	32.522	< 0.001
Students	107 (9.3)	72 (67.3)	35 (32.7)		
Free employment	256 (22.3)	143 (55.9)	113 (44.1)		
Civil servant/private employee	710 (62.0)	347 (48.9)	363 (51.1)		
*Education*					
Master degree and above	100 (8.7)	51 (51.0)	49 (49.0)	5.688	0.224
Bachelor	508 (44.3)	270 (53.1)	238 (46.9)		
College	252 (22.0)	127 (50.4))	125 (49.6)		
High school or technical secondary school	189 (16.5)	113 (59.8)	76 (40.2)		
Middle school/primary school	97 (8.5)	58 (59.8)	39 (40.2)		
*Living status*					
Living alone	331 (28.9)	160 (48.3)	171 (51.3)	6.036	0.014
Living with others	815 (71.1)	459 (56.3)	356 (43.7)		
*Monthly income (RMB)*					
≤ 4000	476 (41.5)	310 (65.1)	166 (34.9)	41.575	< 0.001
> 4000	670 (34.1)	309 (46.1)	361 (53.9)		
*Being with lover or not*					
Yes	642 (56.0)	327 (50.9)	315 (49.1)	5.573	0.018
No	504 (44.0)	292 (57.9)	212 (42.1)		
*History of substance abuse (drugs, aphrodisiacs, *etc*.) in the past 12 months*					
Yes	108 (9.4)	57 (52.8)	51 (47.2)	0.073	0.787
No	1038 (90.6)	562 (54.1)	476 (45.9)		
*Other chronic diseases except HIV*					
Yes	297 (25.9)	195 (65.7)	102 (34.3)	21.878	< 0.001
No	849 (74.1)	424 (49.9)	425 (50.1)		
*The results of the last physical examination*					
Normal	880 (76.8)	468 (53.2)	412 (46.8)	1.057	0.034
Abnormal	266 (23.2)	151 (56.8)	115 (21.8)		
*HIV disclosure*					
Telling family members	602 (52.5)	313 (52.0)	289 (48.0)	2.085	0.149
Family members were not told	544 (47.2)	306 (56.3)	238 (43.8)		
Telling friends	498 (43.5)	252 (50.6)	246 (49.4)	4.127	0.042
Didn't tell a friend	648 (56.5)	367 (56.6)	281 (43.4)		
Telling others	558 (48.7)	288 (51.6)	270 (48.4)	2.524	0.112
No one else was told	588 (51.3)	331 (56.3)	257 (43.7)		
*Anxiety*					
Yes	427 (37.3)	324 (75.9)	103 (24.1)	130.985	< 0.001
No	719 (62.7)	295 (41.0)	424 (59.0)		
*Depression*					
Yes	534 (46.6)	378 (70.8)	156 (29.2)	113.251	< 0.001
No	612 (53.4)	241 (39.4)	371 (60.6)		
*Social support*					
Low	156 (13.6)	109 (69.9)	47 (30.1)	55.339	< 0.001
Medium	660 (57.6)	385 (58.3)	275 (41.7)		
High	330 (28.8)	125 (37.9)	205 (62.1)		

### Risk factors of suicidal ideation in PLWH

Binary logistic regression analysis results showed that the PLWH who with short time for HIV positive diagnosis (*aOR* (adjusted odd ratio) = 1.754, 95%CI(confidence interval):1.338–2.299), low monthly income (*aOR* = 1.515, 95%*CI*:1.098–2.092), other chronic diseases except HIV (*aOR* = 1.555, 95%*CI*:1.134–2.132), irregular lovers (*aOR* = 1.369, 95%*CI*:1.021–1.837), anxiety (*aOR* = 2.711, 95%*CI*:1.767–4.161), depression (*aOR* = 1.614, 95%*CI*:1.078–2.417), low PSSS (*aOR* = 2.139, 95%*CI*:1.345–3.399) had high risk of suicide ideation. (Table 2).

**Table 2:** Multivariate analysis of suicidal ideation in PLWH according to logistic analysis

Variables	*β*	SE	Wald*χ*^2^	aOR(95%CI)	*P*
*HIV positive diagnosis time*					
≤ 35 months	0.562	0.138	16.539	1.754 (1.338–2.299)	< 0.001
> 36 months				1	
*Monthly income (RMB)*					
≤ 4000	0.416	0.164	6.389	1.515 (1.098–2.092)	0.011
≥ 4001				1	
*Other chronic diseases except HIV*					
Yes	0.441	0.161	7.517	1.555 (1.134–2.132)	0.006
No				1	
*Being with lover or not*					
Yes				1	
No	0.314	0.150	4.393	1.369 (1.021–1.837)	0.036
*Anxiety*					
Yes	0.997	0.219	20.837	2.711 (1.767–4.161)	< 0.001
No					
*Depression*					
Yes	0.479	0.206	5.394	1.614 (1.078–2.417)	0.020
No				1	
*PSSS*					
Low	0.760	0.236	10.337	2.139 (1.345–3.399)	0.001
Medium	0.093	0.217	0.183	1.097 (0.717–1.679)	0.669
High				1	

### Relations between suicide ideation, anxiety, depression and PSSS

Social support was negatively correlated with depression, anxiety, and suicidal ideation (*P* < 0.01); Depression was positively correlated with anxiety and suicidal ideation (*P* < 0.01); Anxiety was positively correlated with suicidal ideation (*P* < 0.01) (Table 3).

**Table 3:** Relations between suicide ideation, anxiety, depression and PSSS

	PSSS	PHQ-2	GAD-2	Suicide ideation
PSSS	1			
PHQ-2	− 0.270**	1		
GAD-2	− 0.272**	0.899**	1	
Suicide ideation	− 0.263**	0.750**	0.595**	1

**P < 0.01, which means the relation analyses were significant

### Mediating role of PSSS in anxiety, depression and suicidal ideation

Table 4 showed the results of the mediating role of the PSSS in anxiety, depression and suicide ideation respectively. Firstly, it shows that anxiety of PLWH can directly affect its suicidal ideation, and it can also indirectly affect its suicidal ideation through the reduction of social support. The proportion of intermediary effect in total effect is − 0.261 × − 0.083/0.712 = 30.43%. Secondly, it shows that the depression of PLWH can directly affect its suicide ideation, and also indirectly affect its suicide ideation through the reduction of social support. The proportion of intermediary effect in total effect is − 0.270 × − 0.066/0.750 = 23.76%.

**Table 4:** Tests of the mediating role of PSSS in anxiety, depression and suicidal ideation

	Independent variable	Dependent variable	Regression equation	*β*	SE	*t*	*P*
*Test 1: the mediating role of PSSS in anxiety and suicidal ideation*							
Step 1	Anxiety	Suicide ideation	Y = 0.712X	0.712	0.21	34.296	< 0.001
Step 2	Anxiety	PSSS	M = − 0.261X	− 0.261	0.29	− 9.132	< 0.001
Step 3	Anxiety	Suicide ideation	Y = 0.690X	0.690	0.21	32.301	< 0.001
	PSSS		− 0.083 M	− 0.083	0.21	− 3.889	< 0.001
*Test 2: the mediating role of PSSS in depression and suicidal ideation*							
Step 1	Depression	Suicide ideation	Y = 0.750X	0.750	0.020	38.307	< 0.001
Step 2	Depression	PSSS	M = − 0.270X	− 0.270	0.028	− 9.471	< 0.001
Step 3	Depression	Suicide ideation	Y = 0.732X	0.732	0.020	36.166	< 0.001
	PSSS		− 0.066 M	− 0.066	0.20	− 3.246	0.001

## Discussion

### Factors influencing suicide ideation among PLWH

In this study, the rate of suicide ideation of PLWH was 54.01% (619/1146) in the past week or the period of their most serious depression, which should be pay attention. PLWH with short HIV positive diagnosis time, low monthly income, other chronic diseases except HIV, unstable lovers, anxiety, depression, and low social support are more likely to have suicidal ideation. Consistent with previous study, HIV infection is highly related to a series of mental health problems, such as depression, anxiety and adaptive disorder [20]. Some PLWH may also have employment difficulties or even affect economic income due to other chronic diseases. Good social support may enable PLWH to better cope with the negative impact caused by pressure [21]. However, when faced with the pressure of stigma, external discrimination, and problem telling, PLWH often chooses to hide their infected identity, so that it cannot take advantage of social support from family, friends and lovers [22]. As well as we know, newly diagnosed HIV is a major stress event (even a traumatic event) for individuals. Along with the passage of time and the progress of ART, many PLWH could gradually recover CD4 cells and control the viral load. Some infected people will gradually adapt to the identity of HIV infected people, and they take the initiative to inform their families, friends or loved ones, which help them get more social support and recover their mental and physical health gradually [23].

### Suicide ideation of PLWH relates to their depression, anxiety and low PSSS

Results of this study showed that the PLWH with suicidal ideation was more likely to have anxiety and depression, along with less perceived social support, which was consist with other studies. According to the interpersonal relationship theory of suicide [24], one person who wants to die by suicide must have three elements: frustrated sense of belonging, perceived burden and learned suicide ability. First, individuals who think that they have less social support from family and friends were with a relatively weak sense of belonging generally. Second, due to the incurability and the particular infection way of AIDS, the PLWH always suffer from heavy pressure, shame and social discrimination than people of other diseases [25, 26]. Tangney and Dearing have reported that the shame is a kind of self-conscious emotion related to impotence and inferiority [27], which leads to negativity, withdrawal, insignificance, worthlessness and powerlessness of PLWH. In this state, individuals are prone to have a sense of burden. Third, if individuals are in negative emotions without correct identification, description and regulation, people are prone to psychosomatic diseases, depression, anxiety disorder and so on [28]. All these factors may lead to suicidal ideation.

Besides, PLWH with anxiety, depression and less perceived family support were more likely to occur suicidal ideation. On the one hand, PLWH with high shame experience is prone to be self-criticism, depression and social anxiety excessively [29, 30], which results in the reducing of their seeking social support from friends and relatives. Then, family support has become the key social support for PLWH. If the PLWH feels insufficient family support, they may develop the idea of suicide ideation after their enhancing negative emotional experience, self-shame, anxiety and depression. On the other hand, the low family support of PLWH may lead to their alienation of family and the enhancement of their shame experience, which further results in the difficulty for the family to have close associations with them and provide realistic and psychological support. Therefore, there is a need to pay more attention to the anxiety, depression and social support when preventing suicide among the PLWH.

### The mediating effect of PSSS between anxiety, depression and suicide ideation

The mediating effect analysis showed that the social support plays a partial mediating role between anxiety, depression and suicidal ideation. Firstly, anxiety and depression can directly increase the suicide ideation of PLWH. Secondly, anxiety and depression can also indirectly increase the suicide ideation of PLWH by leading to low social support; on the contrary, high social support will reduce the effect of anxiety and depression on suicide ideation of PLWH.

This results indicating that there are two ways to reduce the suicide ideation of PLWH: 1) Measures should be taken to reduce anxiety and depression of PLWH, such as popularization the health education of AIDS disease, reducing social discrimination and ostracism of AIDS patients. 2) To improve the social support of PLWH. We need to pay more attention to improve the social support of AIDS patients, so as to reduce their suicide ideation of PLWH. Previous studies have shown that it could alleviate individual stress by providing emotional support, material help and other forms of social support [31]. PLWH is often difficult to obtain social support directly from relatives and friends, so the psychological support and care provided by medical staff for AIDS patients plays a very important role. Therefore, it is very feasible for health service personnel (especially AIDS prevention and control personnel) taking their advantages of profession and access to provide sufficient and sustainable psychological care and support for PLWH coping with AIDS related pressure and maintaining better life quality.

Overall, according to the suicide ideation surveys of PLWH around the world, the number of PLWH with suicide ideation is increasing and getting younger [32, 33]. Moreover, the MSM should be paid more attention among the PLWH for their higher psychological pressure than others. Due to the significantly protection effect of the high perceived family and social support on suicide ideation [34], it would be effective measures to help PLWH reduce their pressure and psychological burden by eliminating social discrimination, mobilizing social and family support actively, and establishing social support model and so on.

### Limitations

The study had several limitations: (1) The differences of investigation environment between WeChat and face-to-face might lead little bias in this study. For instance, some body languages of respondents were difficult to be observed by the investigation way of WeChat. (2) We cannot exclude the bias from the self-reported design, and we cannot infer the causation in this study for its cross-sectional design. (3) The concept of suicide ideation is different from suicide, so studies on suicide ideation could not fully guide the intervention of suicide crisis. (4) limited to the online survey design, some indicators such as physiological indicators could not be obtained in our this study. In the future, case–control study and psychological anatomy might be used to further explore the relationships between suicide and depression, anxiety and social support in PLWH with suicidal behavior.

## Conclusion

The rate of suicide ideation among PLWH is high. Suicide ideation of PLWH are related to their depression, anxiety and perceived social support, and social support plays a partial mediating role between anxiety, depression and suicidal ideation. Anxiety, depression and perceived social support should be considered into the suicide intervention strategy of PLWH, which provides a new approach for prevention of suicidal ideation in PLWH and should be known widely for people to prevent suicide. Moreover, results from this study could be provided for healthcare providers and other persons who want to design an effective health education programme for the targeted people.

## Data Availability

The datasets used and/or analyzed of this study available from the corresponding author on reasonable request.

## Abbreviations

HIV/AIDS: Human immunodeficiency virus/acquired immune deficiency syndrome
PSSS: Perceived social support scale
BSI-CV: The Beck scale for suicide ideation of Chinese version
GAD-2: The generalized anxiety disorder scale
PHQ-2: The patient health questionnaire-2
OR: Odd ratio
CI: Confidence interval
PLWH: People living with HIV
ART: Antiretroviral therapy
CDC: Center for disease control and prevention
STI: Sexually transmitted infections
WHO: World Health Organization
SPSS: Statistical program for social sciences
SD: Standard deviation
MSM: Male have sex with male
KMO: Kaiser–Meyer–Olkin
AUC: Area under the curve
ICC: Intraclass correlation coefficient
